# Thyroid Hormone Changes in Early Pregnancy Along With the COVID-19 Pandemic

**DOI:** 10.3389/fendo.2020.606723

**Published:** 2020-12-07

**Authors:** Ting-Ting Lin, Chen Zhang, Han-Qiu Zhang, Yu Wang, Lei Chen, Cindy-Lee Dennis, Hefeng Huang, Yan-Ting Wu

**Affiliations:** ^1^ The International Peace Maternity and Child Health Hospital, School of Medicine, Shanghai Jiao Tong University, Shanghai, China; ^2^ Shanghai Key Laboratory of Embryo Original Diseases, Shanghai, China; Chinese Maternal and Child Health Association, Beijing, China; ^3^ Lawrence S. Bloomberg Faculty of Nursing, University of Toronto, Ontario, Canada

**Keywords:** thyroid, early pregnancy, COVID-19, anxiety, depression

## Abstract

**Purpose:**

COVID-19 (Coronavirus Disease 2019) was first reported in December 2019 and quickly swept across China and around the world. Levels of anxiety and depression were increased among pregnant women during this infectious pandemic. Thyroid function is altered during stressful experiences, and any abnormality during early pregnancy may significantly affect fetal development and pregnancy outcomes. This study aimed to determine whether the COVID-19 pandemic induces thyroid hormone changes in early pregnant women.

**Methods:**

This study comprised two groups of pregnant women in Shanghai in their first trimester – those pregnant women before the COVID-19 outbreak from January 20, 2019, to March 31, 2019 (Group 1) and those pregnant during the COVID-19 outbreak from January 20, 2020, to March 31, 2020 (Group 2). All women were included if they had early pregnancy thyrotropin (TSH), free triiodothyronine (FT3), free thyroxine (FT4), total triiodothyronine (TT3), and total thyroxine (TT4) concentrations, thyroid peroxidase (TPO) antibody or thyroglobulin antibody (TgAb) available and did not have a history of thyroid diseases or received thyroid treatment before or during pregnancy. We used propensity score matching to form a cohort in which patients had similar baseline characteristics.

**Results:**

Among 3338 eligible pregnant women, 727 women in Group 1 and 727 in Group 2 had similar propensity scores and were included in the analyses. Pregnant women in Group 2 had significantly higher FT3 (5.7 vs. 5.2 pmol/L, P<0.001) and lower FT4 (12.8 vs. 13.2 pmol/L, P<0.001) concentrations compared with those in Group 1. Pregnant women in Group 2 were more likely to develop isolated hypothyroxinemia (11.6% vs. 6.9%, OR, 1.75 [95% CI, 1.20–2.53], *P*=0.003) than those in Group 1 but had a significantly lower risk of TgAb positivity (12.0% vs. 19.0%, OR, 0.58 [95% CI, 0.43–0.78], *P*<0.001).

**Conclusion:**

Pregnant women in their first trimester in Shanghai during the COVID-19 outbreak were at an increased risk of having higher FT3 concentrations, lower FT4 concentrations, and isolated hypothyroxinemia. The association between thyroid hormones, pregnancy outcomes, and the COVID-19 outbreak should be explored further.

## Introduction

COVID-19 (Coronavirus Disease 2019), caused by SARS-Cov-2 (Severe Acute Respiratory Syndrome Coronavirus 2) virus, is a highly infectious disease with a significant mortality rate and limited effective treatment (1). The outbreak was first reported in December 2019 in Wuhan, China, and since then, the number of cases has continued to escalate exponentially worldwide (2). The severity of COVID-19 was underestimated before it was officially confirmed as a type B infectious disease by the National Health Council, and the nation took heightened action to fight against it on January 20, 2020 (3). Since then, the number of diagnoses and deaths has grown rapidly internationally with significant epidemic prevention work implemented (4). Due to the uncertainty and low predictability of COVID-19, depression and anxiety symptoms have been common among the general population (5–7).

Pregnant women are also affected by the COVID-19 pandemic due to safety concerns for their fetuses (8, 9). In a case-control study, anxiety levels among pregnant women during the SARS outbreak were significantly higher than levels pre-SARS outbreak (8). A recent cross-sectional study conducted in pregnant women during the COVID-19 pandemic found that half of the participants indicated that the COVID-19 outbreak had serious psychological effects, with two-thirds reporting higher than normal levels of stress (9). Almost half of the women expressed high anxiety due to concerning potential vertical transmission of this disease (9). The negative psychological impact caused by COVID-19 was especially highlighted among women in their early pregnancies (9). This kind of stress and anxiety may have a significant physiological impact on pregnant women.

Thyroid hormones regulate various physiological processes related to pregnancy, such as the development and function of the placenta, fetal growth, and the expression of neuropeptides at the onset of labor (10–12). Overt thyroid diseases, such as hyperthyroidism and hypothyroidism, occur in approximately 0.5% and 0.05% of pregnant women, respectively and are associated with adverse outcomes for pregnant women and their fetuses (13, 14). Compared with overt thyroid diseases, thyroid dysfunctions such as subclinical hyperthyroidism and isolated hypothyroxinemia and thyroid autoimmune diseases have higher incidences (14) and have been associated with an increased risk for preeclampsia, spontaneous abortion, preterm delivery, low birth weight, and intrauterine growth retardation (IUGR) (15). Thus, the timely detection and treatment of pregnant women with thyroid dysfunction is clinically important (16). Few studies have focused on the physiological and psychological well-being of pregnant women during an infectious disease outbreak to understand the development of subclinical diseases that also need clinical management. The purpose of this study was to examine whether the COVID-19 outbreak was independently associated with thyroid hormone fluctuation and an increased risk of thyroid dysfunction in early pregnancy.

## Materials and Methods

### Sample Collection

This retrospective cohort study was conducted in the International Peace Maternity and Child Health Hospital (IPMCH), which is a university-affiliated hospital and accounts for approximately 20% of births in Shanghai, China. As a part of standard antenatal care, all pregnant women underwent thyroid assessment in their first trimester and were divided into two groups. On January 20, 2020, COVID-19 was officially classified as a Type B infectious disease by the National Health Commission and thus defined as the start date of public awareness. Those pregnant women who underwent thyroid assessment between January 20, 2019, and March 31, 2019, were classified as the pre–COVID-19 epidemic group (Group 1), and those pregnant women screened between January 20, 2020, and March 31, 2020, were classified as the during-COVID-19 epidemic group (Group 2). A total of 3038 women in Group 1 and 2082 women in Group 2 were enrolled, all of whom had thyrotropin (TSH), free thyroxine (FT4), free triiodothyronine (FT3), TPOAb or TgAb concentrations available and had no COVID-19 symptoms such as fever, cough, and myalgia symptoms (1). Women were excluded if they were not in their first trimester, underwent *in vitro* fertilization, had twin pregnancies, or had a history of thyroid disease or TPOAb positivity. Ethics approval was obtained by the institutional review board (No. GKLW2019-51).

### Exposure and Outcomes

When pregnant women come to the hospital for prenatal care, all data, including sociodemographic and clinical data, were collected by doctors and nurses and recorded in the electronic medical file. Thyroid hormones, including TSH, FT3, FT4, TT3, TT4, TPOAb, and TgAb, were measured by taking fasting blood samples from the cubital vein and centrifugation within 6 hours to separate the serum, which was then detected with the Architect i2000 immunoassay (Abbott, Chicago, USA) during the whole study period. The lower limits of detection and the intra- and interassay coefficients of variation were 0.0038 mIU/L and 1.6% and 3.59%, respectively, for TSH; 1.54 pmol/L and 2.97% and 4.03%, respectively, for FT3; 0.6200 pmol/L and 1.9% and 4.01%, respectively, for FT4; 0.38 nmol/L and 2.3% and 4.07%, respectively, for TT3; 0.62 pmol/L and 1.9% and 4.01%, respectively, for TT4; 0.5 IU/ml and 10% and 10%, respectively, for TPOAb; and 0.31 IU/ml and 20% and 20%, respectively, for TgAb.

Outcomes included thyroid function measurements (thyrotropin (TSH), free triiodothyronine (FT3), free thyroxine (FT4), total triiodothyronine (TT3), and total thyroxine (TT4) concentrations, thyroid peroxidase (TPO) antibody and thyroglobulin antibody (TgAb)). Subclinical thyroid diseases were defined according to the cohort-specific 2.5th and 97.5th population percentiles for TSH, FT3, FT4, TT3 and TT4 after exclusion of TPO antibody-positive women (17). TPO antibodies ≥5.61 IU/ml were considered positive. TgAbs ≥4.11 IU/ml were considered positive. Subclinical hyperthyroidism was defined as a TSH concentration below the 2.5th percentile and an FT4 concentration within the normal range (2.5th–97.5th percentile). Subclinical hypothyroidism was defined as a TSH concentration above the 97.5th percentile and an FT4 concentration within the normal range. Overt hyperthyroidism was defined as a TSH concentration below the 2.5th percentile and an FT4 concentration above the 97.5th percentile. Overt hypothyroidism was defined as a TSH concentration above the 97.5th percentile and an FT4 concentration below the 2.5th percentile. Isolated hypothyroxinemia was defined as an FT4 concentration below the 2.5th percentile and a TSH concentration within the normal range. Elevated T3 was defined as a TT3 concentration above the 97.5th percentile. Low T3 was defined as a TT3 concentration below the 2.5th percentile.

### Sociodemographic Outcomes

Maternal age, educational level, gravida, and body mass index (BMI) were collected in addition to smoking and drinking status, gestational weeks, and previous history of thyroid disease. We also collected paternal age and education. Maternal and paternal age was reported at the time of thyroid function tests and was categorized as 18 to 29, 30 to 39 and > 39 years. Maternal and paternal educational levels were defined as the years of education after graduation from primary school and were categorized as < 6 years (low), 6 to 10 years (middle) and > 10 years (high). Gravida was defined as the total number of pregnancies, including live births, stillbirths and abortions. BMI was calculated as weight in kilograms divided by height in meters squared and categorized as low weight (<18.5), normal weight (18.5–23.9), overweight (24.0–27.9), and obese (≥28.0). Maternal smoking was self-reported and categorized as nonsmoker, past smoker or current smoker. Maternal drinking was similarly categorized. Weeks gestation was established based on measurement of the fetal crown–rump length (CRL) (18). A previous history of thyroid disease was defined as self-reported hyperthyroidism, hypothyroidism, thyroid nodules that underwent thyroidectomy, and thyroid autoimmune diseases.

### Statistical Analysis

Considering the difference in baseline characteristics between the two groups of participants (**Table 1**), we used propensity score matching to regroup a cohort of 1454 first trimester pregnant women with similar baseline characteristics. The propensity score is the conditional probability of having a specific exposure level (Group 1 and Group 2) under a given set of baseline covariates (19, 20). A multivariate logistic regression model was used to estimate propensity scores, and all baseline characteristics listed in **Table 1** were included as covariates, using a 1:1 matching protocol with no replacement for matching with a caliper width equal to 0.01 and evaluating the P values of all baseline covariates before and after matching to assess whether they are balanced. Standard differences of less than 5% indicate a relatively small imbalance. All variables in the matched cohort are without missing data.

**Table 1:** Baseline characteristics before and after propensity-score matching.

Assessment characteristics	Before matching			After matching		
	Group 1N = 2,012	Group 2N = 1,326	P value	Group 1 N = 727	Group 2 N = 727	P value
**Maternal Demographics**						
Age, median (Q1–Q3) in years	30 (28–32)	31 (28–33)	<0.001	30 (28–33)	30 (28–33)	0.52
Age distribution						
18–29	937/2,012 (46.6)	499/1,326 (37.6)	<0.001	297 (40.9)	289 (39.8)	0.52
30–39	1,046/2,012 (52.0)	786/1,326 (59.3)		417 (57.4)	420 (57.8)	
>39	29/2,012 (1.4)	41/1,326 (3.1)		13 (1.8)	18 (2.5)	
Weeks gestational at blood sampling, median (95% range)	12.7 (12.3–13.1)	12.1 (11.4–12.9)	<0.001	12.4 (12.0–13.0)	12.4 (12.0–12.9)	0.78
Body mass index, median (Q1-Q3), kg/m^2^	20.8 (19.3–22.7)	20.9 (19.3–−22.9)	0.51	20.9 (19.3–−22.9)	20.9 (19.3–−23.0)	0.91
Body mass index distribution						
Underweight	251/1,937 (13.0)	126/982 (12.8)	0.15	95 (13.1)	99 (13.6)	0.74
Normal	1391/1,937 (71.8)	700/982 (71.3)		514 (70.7)	508 (69.9)	
Overweight	244/1,937 (12.6)	115/982 (11.7)		95 (13.1)	87 (12.0)	
Obesity	51/1,937 (2.6)	41/982 (4.2)		23 (3.2)	33 (4.5)	
Gravida, median (Q1–Q3)	1 (1–2)	2 (1–2)	0.08	2 (1–2)	1 (1–2)	0.50
Smoking status						
Nonsmoker or past smoker	1,916/1,923 (99.6)	969/977 (99.2)	0.11	725 (99.7)	722 (99.3)	0.45
Current smoker	7/1923 (0.5)	8/977 (0.8)		2 (0.3)	5 (0.7)	
Drinking status						
Nondrinker or past drinker	1,871/1,923 (97.3)	963/977 (98.6)	0.03	713 (98.1)	716 (98.5)	0.55
Current drinker	52/1,923 (2.7)	14/977 (1.4)		14 (1.9)	11 (1.5)	
Educational level						
Low	140/1,935 (7.2)	74/981 (7.5)	0.11	41 (5.6)	52 (7.2)	0.64
Middle	1,411/1,935 (72.9)	678/981 (69.1)		530 (72.9)	517 (71.1)	
High	384/1,935 (19.8)	229/981 (23.3)		156 (21.5)	158 (21.7)	
**Paternal Demographics**						
Age, median (Q1–Q3) in years	32 (29–35)	32 (30–36)	<0.001	32 (30–35)	32 (30–35)	0.90
Age distribution						
18–29	540/1,885 (28.6)	202/948 (21.3)	<0.001	163 (22.4)	161 (22.2)	0.96
30–39	1,222/1,885 (64.8)	669/948 (70.6)		505 (69.5)	510 (70.2)	
>39	123/1,885 (6.5)	77/948 (8.1)		59 (8.1)	56 (7.7)	
Educational level						
Low	297/3,211 (9.2)	40/454 (8.8)	0.19	44 (6.1)	52 (7.2)	0.96
Middle	2,216/3,211 (69.0)	301/454 (66.3)		509 (70.0)	494 (68.0)	
High	698/3,211 (21.7)	113/454 (24.9)		174 (23.9)	181 (24.9)	

Descriptive analysis of normally distributed continuous variables is expressed as the mean and standard deviation (SD). For nonnormally distributed variables, we use the median and interquartile range (IQR); categorical variables are expressed in proportion and percentage. We used the Mann-Whitney U test to compare the median difference of continuous variables and the chi-square test to compare the difference in proportion. To perform a univariate analysis of the association between thyroid dysfunction and all other factors, a chi-square test was used (if the number in the cell was less than 5, Fisher’s two-sided exact test was used). In a multivariate logistic regression model, logistic regression analysis was performed on the relationship between (subclinical) thyroid diseases and all those variables with a P value <0.1 in univariate analysis. Sensitivity analysis and subgroup analysis were used to evaluate the difference in thyroid hormones and (subclinical) thyroid diseases in each subgroup between the matched Group 1 and Group 2 to identify potential bias. A two-sided P value of less than 0.05 was considered statistically significant. All statistical tests were conducted using SPSS version 24.0 and R version 3.6 (*Packages forestplot, ggplot2*).

## Results

### Sample Characteristics

**Figure 1:**
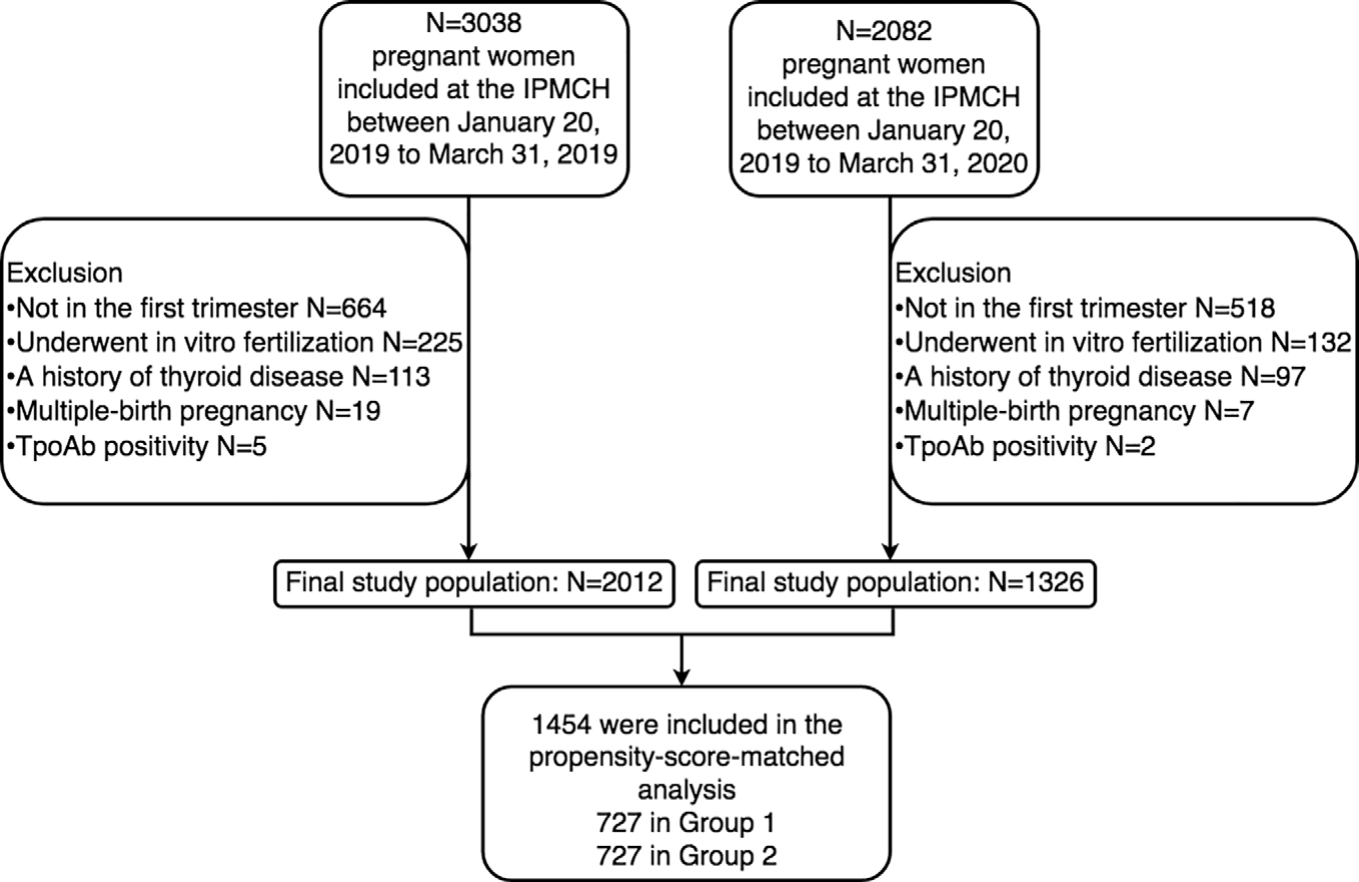
Flowchart illustrating study population selection and data availability.

After exclusion, a total of 3338 pregnant women participated in the study, with 2012 in Group 1 and 1326 in Group 2 (**Figure 1**). **Table 1** shows the comparisons of the baseline characteristics between the two groups before and after propensity-score matching. The majority of women overall were between 30 and 39 years old (n =1832, 54.9%), nondrinkers (n =2834, 97.7%), and nonsmokers (n =2885, 99.5%). Compared with Group 1, Group 2 was more likely to be older (31 vs 30 years old, P<0.001), earlier in pregnancy (i.e., lower weeks gestation) (12.1 vs 12.7 weeks, P<0.001) and less likely to be a current drinker (1.4% vs. 2.7%, P=0.03). With propensity score matching, 727 women exposed to the COVID-19 outbreak (Group 2) were matched with 727 women in Group 1. After matching, the P values of all variables were greater than 0.05, indicating that no significant differences existed between the two groups (**Table 1**).

### Thyroid Hormone Concentrations Among Early Pregnant Women

Women in Group 2 who were exposed to the COVID-19 outbreak had significantly higher FT3 (5.7 vs. 5.2 pmol/L, P<0.001) and lower FT4 (12.8 vs. 13.2 pmol/L, P<0.001) concentrations than those in Group 1. There were no significant differences between the two groups in the concentrations of TSH (1.32 vs. 1.28 mIU/L, P=0.45), TT3 (2.0 vs. 2.0 nmol/L, P=0.73) and TT4 (140.2 vs. 140.4 nmol/L, P=0.56) (**Table 2**) (**Figure S1**).

**Table 2:** Thyroid hormone concentrations in the propensityscore-matched cohort.

	Group 1	Group 2	P value
TSH, mIU/L (median, 95% range)	1.28 (0.03–4.15)	1.32 (0.02–3.9)	0.45
Free thyroxine 3 (FT3), pmol/L (median,95% range)	5.2 (4.2–6.6)	5.7 (4.3–7.4)	<0.001
Free thyroxine 4 (FT4), pmol/L (median,95% range)	13.2 (11–16.5)	12.8 (10.7–16.9)	<0.001
Total thyroxine 3 (TT3), nmol/L (median,95% range)	2.0 (1.4–2.8)	2.0 (1.5–2.7)	0.73
Total thyroxine 4 (TT4), nmol/L (median,95% range)	140.4 (100.2–197.3)	140.2 (100.3–192.3)	0.56

### Risk of Thyroid Diseases Among General Early Pregnant Women

Univariate analysis found that factors associated with subclinical thyroid disease included weeks gestational, gravida, maternal BMI, paternal age and educational levels, and cohort group (Group 1 vs Group 2) (**Table S1**). To examine the risk of developing subclinical thyroid disease, logistic regression was performed with results suggesting that exposure to the COVID-19 outbreak increased a woman’s risk of isolated hypothyroxinemia (11.6% vs. 6.9%, OR, 1.75 [95% CI, 1.20–2.54], *P*=0.003) and decreased their risk of TgAb positivity (12.0% vs. 19.0%, OR, 0.58 [95% CI, 0.43–0.78], *P*<0.001) (**Table 3**). Conversely, there was no significant difference between the two groups in the risk of subclinical hyperthyroidism (3.0% vs. 1.9%, OR, 1.60 [95% CI, 0.81–3.15], *P*=0.18), subclinical hypothyroidism (2.6% vs. 4.4%, OR, 0.58 [95% CI, 0.33–1.04], *P*=0.07) and overt hyperthyroidism (0.3% vs. 0.6%, OR, 0.51 [95% CI, 0.09–2.78], *P*=0.43) (**Table 3**) (**Figure S2**).

**Table 3:** Risk of thyroid diseases in the propensity score-matched cohort.

(Subclinical) Thyroid diseases	Group 1n(%)	Group 2n(%)	Odds Ratio (95%)	Adjusted Odds Ratio (95% CI)	P value
Subclinical hyperthyroidism	14 (1.9)	22 (3.0)	1.59 (0.81–3.13)	1.60 (0.81–3.15)a	0.18
Subclinical hypothyroidism	32 (4.4)	19 (2.6)	0.58 (0.33–1.04)	0.58 (0.33–1.04)	0.07
Overt hyperthyroidism	4 (0.6)	2 (0.3)	0.50 (0.10–2.73)	0.51 (0.10–2.78)b	0.43
Isolated Hypothyroxinemia	50 (6.9)	84 (11.6)	1.77 (1.23–2.55)	1.75 (1.20–2.54)c	0.003
Thyroglobulin antibody positivity	138 (19.0)	87 (12.0)	0.58 (0.43–0.78)	0.58 (0.43–0.78)	<0.001
Elevated T3	50 (6.9)	35 (4.8)	0.69 (0.44–1.07)	0.37 (0.07–1.91)d	0.23
Low T3	5 (0.7)	2 (0.3)	0.40 (0.08–2.06)	0.67 (0.43–1.05)e	0.08

The table shows the number and proportion of pregnant women with (subclinical) thyroid disease during early pregnancy in Group 1 and Group 2. The adjusted odds ratio was calculated in the multivariate logistic regression model by including all confounding factors with a P value <0.1 in univariate analysis. a: Paternal age distribution is included as cofounding factor in logistic regression model; b: Gravida is included as cofounding factor in logistic regression model; c: Maternal BMI is included as cofounding factor in logistic regression model; d: BMI distribution is included as cofounding factor in logistic regression model e: Maternal BMI, paternal educational level, maternal age and age distribution, weeks gestational are included as cofounding factor in logistic regression model.

### Sensitivity Analysis

**Figure 2:**
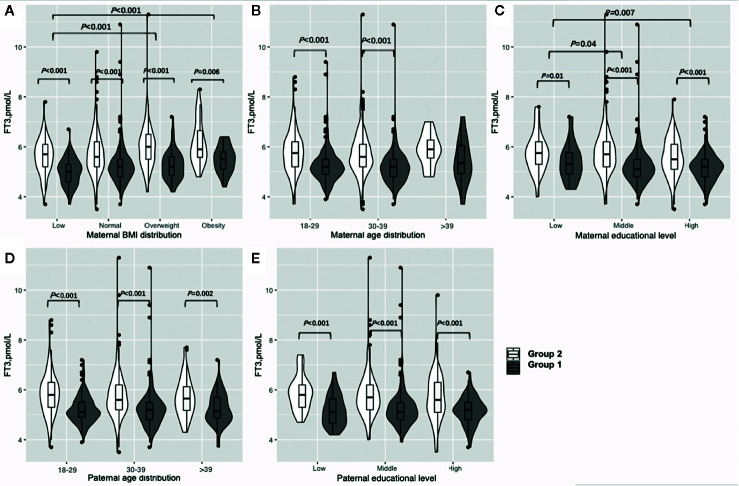
Subgroup analysis of FT3 concentration in the Propensity-Score-Matched Cohort. Figure shows the FT3 concentration in different subgroup of Group 1 and Group 2. The violin diagram is a combination of box plot and density plot. The thick black bar in the middle of the boxplot represents the median and quartile range, and the black line extending up and down represents the 95% confidence interval. The black dots on the black line indicate data values that exceed the 95% confidence interval. The outer contour indicates the distribution of the data. **(A)** Maternal BMI distribution. **(B)** Maternal age distribution. **(C)** Maternal educational level. **(D)** Paternal age distribution. (E) Paternal educational level.

**Figure 2** shows the results of the FT3 subgroup analysis, which indicated that women in early pregnancy exposed to the COVID-19 outbreak who were overweight or obese preconceptionally had a higher FT3 concentration (overweight: 6 vs 5.2 pmol/L, P<0.001, obesity: 5.9 vs. 5.5, P=0.006) (**Figure 2A**, **Table S2**). Furthermore, exposure to the COVID-19 outbreak could elevate FT3 concentration in all subgroups except women over 40 years old (**Figures 2B, D, E**, **Table S2**). A lower maternal education level was associated with higher FT3 concentrations (low vs. middle vs. high: 5.6 vs. 5.4 vs. 5.3 pmol/L, P=0.03) when pooled data from two groups.

**Figure 3:**
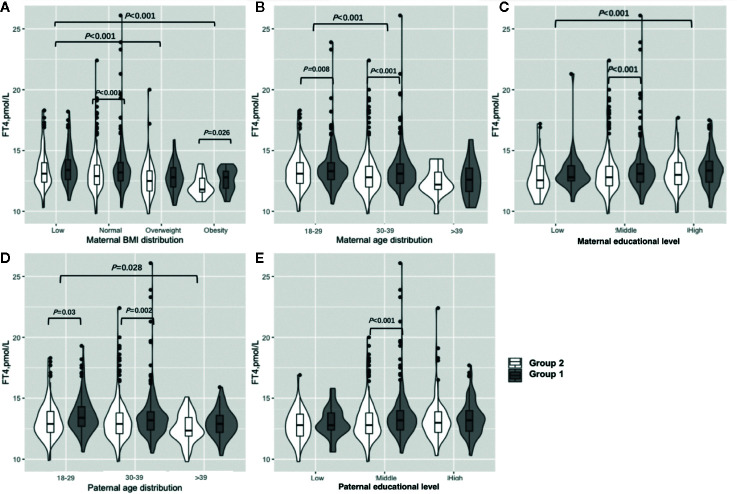
Subgroup analysis of FT4 concentration in the Propensity-Score-Matched Cohort. Figure shows the FT4 concentration in different subgroup of Group 1 and Group 2. The violin diagram is a combination of box plot and density plot. The thick black bar in the middle of the boxplot represents the median and quartile range, and the black line extending up and down represents the 95% confidence interval. The black dots on the black line indicate data values that exceed the 95% confidence interval. The outer contour indicates the distribution of the data. **(A)** Maternal BMI distribution. **(B)** Maternal age distribution. **(C)** Maternal educational level. **(D)** Paternal age distribution. **(E)** Paternal educational level.

**Figure 3** shows the results of the FT4 subgroup analysis. Women in early pregnancy exposed to the COVID-19 outbreak who were overweight or obese preconceptionally had a lower FT4 concentration (overweight: 12.5 vs 12.8 pmol/L, P=0.23, obesity: 11.8 vs. 12.8, P=0.03) (**Figure 3A**, **Table S3**). Those who were less than 30 years old and between 30 and 39 years old had significantly lower FT4 concentrations (< 30: 13.1 vs. 13.3 pmol/L, P=0.008, 30–39: 12.8 vs 13.1 pmol/L, P<0.001) (**Figure 3B**, **Table S3**), and women between 30 and 39 had lower FT4 concentrations (12.9 vs. 13.2 pmol/L, P=0.001) in comparison to those under 30 years of age. Furthermore, the FT4 concentration difference was greater between Group 1 and Group 2 among those between 30 and 39 years old compared to women less than 30 years old (−0.3 vs −0.2 pmol/L, P<0.001) (**Figure 3B**, **Table S3**). Women exposed to the COVID-19 outbreak at all education levels had lower FT4 concentrations than Group 1 (12.8 vs. 13.1 pmol/L, P<0.001), although a significant difference was only found at the middle level (**Figure 3C**, **Table S3**). A higher maternal education level was associated with lower FT4 concentrations (low vs. middle vs. high: 12.75 vs. 13.0 vs. 13.2 pmol/L, P=0.01) when pooled data from two groups. The lower concentration of FT4 in Group 2 than in Group 1 was not significant among women with husbands over 40 years old (12.4 vs 12.9 pmol/L, P=0.07) or in low (12.8 vs 12.8 pmol/L, P=0.27) or high (13.0 vs 13.2 pmol/L, P=0.14) educational levels, but it was significant among those whose husbands were under the age of 40 (under 30: 12.9 vs 13.4 pmol/L, P=0.03, 30 to 39: 12.9 vs 13.2 pmol/L, P=0.002) or had a higher educational level (12.8 vs 13.2 pmol/L, P<0.001) (**Figures 3D, E**, **Table S3**).

**Figure 4:**
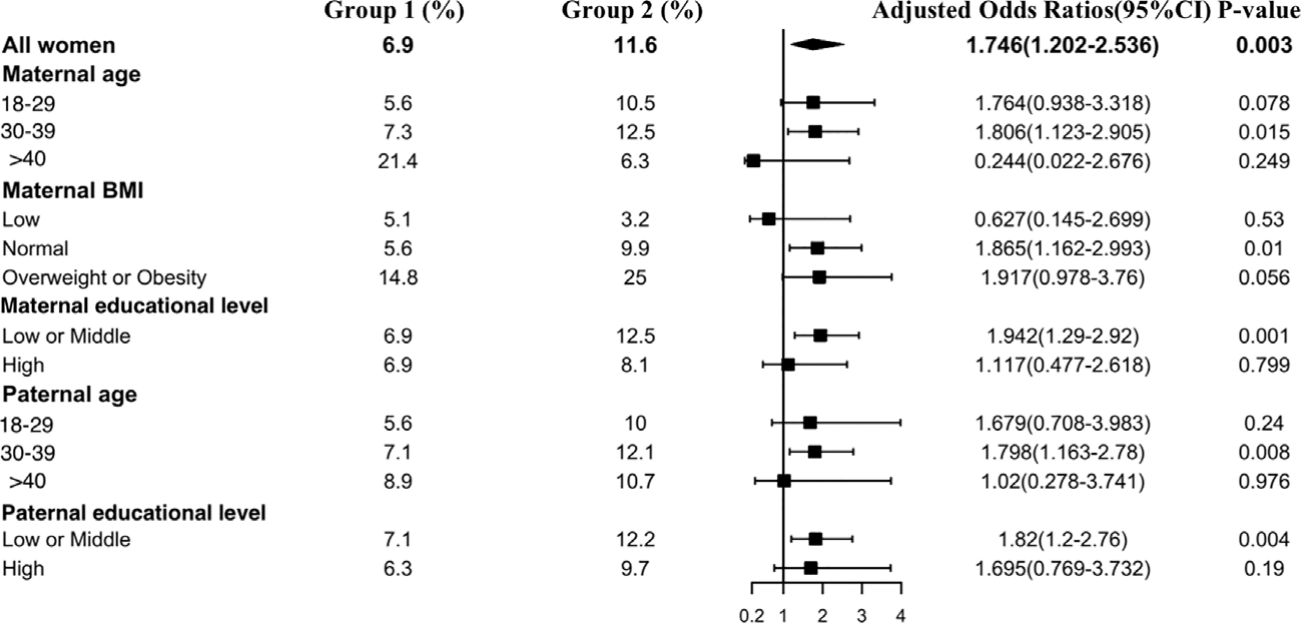
Subgroup analysis of the risk of isolated hypothyroxinemia in the Propensity-Score-Matched Cohort. Figure shows the proportion of pregnant women with isolated hypothyroxinemia in each subgroup of Group 1 and Group 2 (expressed as the number of pregnant women with isolated hypothyroxinemia / the total number of women in this subgroup *100%). The forest chart in the fourth column describes the adjusted odds ratio in each subgroup. The diamond represents the overall adjustment odds ratio. The position of the small rectangle below indicates the point estimate of the adjustment odds ratio of each subgroup. The solid line range indicates the 95% confidence interval of the adjustment risk ratio of each subgroup. Adjusted odds ratio is calculated in the multivariate logistic regression model by including all confounding factors with a P value < 0.1 in univariate analysis.

**Figure 4** shows the results of the isolated hypothyroxinemia subgroup analysis. An increased risk of isolated hypothyroxinemia was found in all subgroups except women over 40 years old or those with an underweight BMI. Women in early pregnancy exposed to the COVID-19 outbreak between 30 and 39 years old had a higher risk of isolated hypothyroxinemia (OR, 1.81 vs. 1.76, P<0.001) in comparison to those under 30 years of age. Women who were overweight or obese preconceptionally had a higher risk of developing isolated hypothyroxinemia in comparison to normal or underweight women exposed to the COVID-19 outbreak (OR 1.92 vs. 1.87 vs. 0.63).

**Figure 5:**
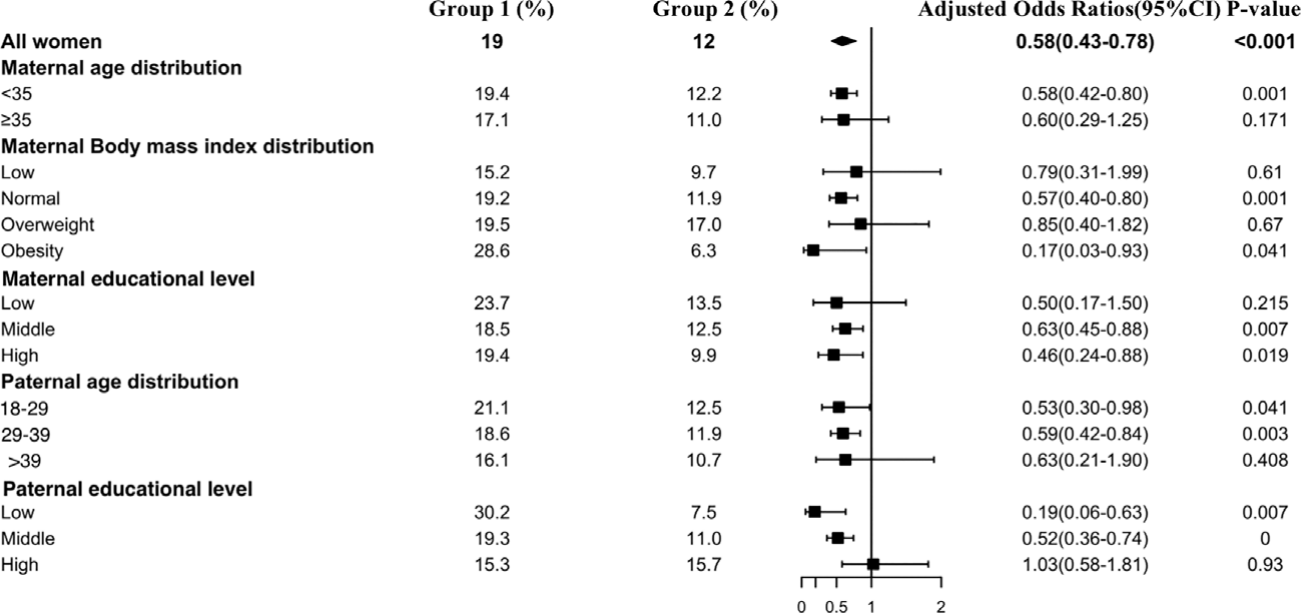
Subgroup analysis of the risk of TgAb positivity in the Propensity-Score-Matched Cohort. Figure shows the proportion of pregnant women with TgAb positivity in each subgroup of Group 1 and Group 2 (expressed as the number of pregnant women with TgAb positivity / the total number of women in this subgroup *100%). The forest chart in the fourth column describes the adjusted odds ratio in each subgroup. The diamond represents the overall adjustment odds ratio. The position of the small rectangle below indicates the point estimate of the adjustment odds ratio of each subgroup. The solid line range indicates the 95% confidence interval of the adjustment risk ratio of each subgroup. Adjusted odds ratio is calculated in the multivariate logistic regression model by including all confounding factors with a P value < 0.1 in univariate analysis.

**Figure 5** shows the results of the TgAb positivity subgroup analysis, which indicated that the risk of TgAb positivity among all subgroups was lower in Group 2 than in Group 1 except at the paternal high educational level. Interestingly, older paternal age was associated with a lower prevalence rate of TgAb positivity in early pregnant women (Group 1: 18–29 vs 29–39 vs >39: 21.1% vs 18.6% vs 16.1%, Group 2: 18–29 vs 29–39 vs >39: 12.5% vs 11.9% vs 10.7%).

After excluding TgAb-positive women, there were 589 in group 1 and 640 in group 2. The results were similar to the population without exclusion. Women in Group 2 who were exposed to the COVID-19 outbreak had significantly higher FT3 (5.7 vs. 5.2 pmol/L, P<0.001) and lower FT4 (12.9 vs. 13.2 pmol/L, P<0.001) concentrations than those in Group 1. There were no significant differences between the two groups in the concentrations of TSH (1.31 vs. 1.24 mIU/L, P=0.37), TT3 (2.0 vs. 2.0 nmol/L, P=0.54) and TT4 (140.9 vs. 141.1 nmol/L, P=0.33) (**Table S4**). In addition, TgAb-negative women exposed to the COVID-19 outbreak had an increased risk of isolated hypothyroxinemia (11.1% vs. 6.5%, OR, 1.77 [95% CI, 1.16–2.69], P=0.008). No significant difference was found in the risk of subclinical hyperthyroidism (3.1% vs. 1.5%, OR, 2.08 [95% CI, 0.94–4.60], P=0.07) or subclinical hypothyroidism (2.5% vs. 3.9%, OR, 0.63 [95% CI, 0.33–1.21], P=0.16) (**Table S5**).

For TgAb-positive women, there were 138 in Group 1 and 87 in Group 2. Those women in Group 2 who were exposed to the COVID-19 outbreak had significantly higher FT3 (5.6 vs. 5.0 pmol/L, P<0.001) and lower FT4 (12.6 vs. 13.2 pmol/L, P=0.03) concentrations than those in Group 1. There were no significant differences between the two groups in the concentrations of TSH (1.47 vs. 1.39 mIU/L, P=0.74), TT3 (2.0 vs. 2.0 nmol/L, P=0.73) and TT4 (136.1 vs. 139.7 nmol/L, P=0.44) (**Table S6**). The COVID-19 outbreak increased the risk of isolated hypothyroxinemia, although there was no significant difference (14.9% vs. 8.7%, OR, 1.93 [95% CI, 0.80–4.64], P=0.14). No significant difference was found in the risk of subclinical hyperthyroidism (2.3% vs. 3.6%, OR, 0.63 [95% CI, 0.12–3.30], P=0.58) or subclinical hypothyroidism (3.4% vs. 6.5%, OR, 0.33 [95% CI, 0.08–1.40], P=0.13) (**Table S7**).

## Discussion

This study found that women in early pregnancy who were exposed to the COVID-19 outbreak had a higher concentration of FT3 and a lower concentration of FT4 in comparison to those not exposed to this pandemic. Furthermore, we found an increased risk of isolated hypothyroxinemia and a lower risk of TgAb positivity among early pregnant women exposed to the COVID-19 outbreak who did not have any previous thyroid diseases after propensity score matching and adjusting for potential confounders, including maternal and paternal age and educational level, gravida status, maternal smoking and drinking status, maternal BMI and weeks gestational.

Infectious disease pandemics will naturally increase anxiety and fear among individuals in society (4). Pregnant women are vulnerable due to additional safety concerns for their fetuses because they also worry that vertical transmission will damage the health of the fetus in addition to their own health (9). A substantial portion of pregnant women overestimated their risk of COVID-19 infection during the outbreak, leading to even higher rates of depression and anxiety than what is typically expected during the antenatal period (9). The depression rate in pregnant women is positively correlated with the number of newly confirmed cases, the number of suspicious cases and the number of deaths per day (21). It is well documented that antenatal depression and anxiety are associated with poor pregnancy outcomes and have negative influences on child development (22–24).

During pregnancy, thyroid hormones of pregnant women undergo significant physiological changes. The concentration of FT4 increases in the early stages of pregnancy and maintains this level until delivery and then rapidly declines postnatally (17). Conversely, FT3 changes little during pregnancy (17). In early pregnancy, the increase in FT4 levels is due to increased placental production of human chorionic gonadotropin (hCG), which leads to a reduction in TSH (11). Although the fetal thyroid begins to develop around the 5th or 6th week of pregnancy, the fetus cannot synthesize thyroid hormone by itself during early pregnancy (25). Thus, the fetal thyroid hormones required for normal neurological development are completely maternally derived. Alterations in maternal FT4 concentration due to significant stress, such as a COVID-19 outbreak, may increase the risk of adverse pregnancy outcomes.

It is currently known that isolated hypothyroxinemia is associated with a higher risk of preterm and very preterm birth (26) and neurocognitive dysplasia in offspring (27, 28). Previous studies have shown a relationship between thyroid antibody positivity and concomitant anxiety or depression disorder, where individuals with autoimmune thyroiditis exhibit an increased chance of developing depression and anxiety (29). However, the relationship between thyroid autoantibodies and preterm delivery is controversial. A prospective cohort study of 120 pregnant women in western European cities reported that those with normal thyroid function but positive TPOAb or TgAb had a significantly increased preterm birth rate (16% vs. 8%, p <0.005) (30). Conversely, another prospective study of 1179 women in Japan did not find an increased risk of preterm birth among TgAb-positive women. The preterm birth rates in the study group and the control group were both extremely low (3% vs. 3.1%), which the researchers suggested was the result of ethnic differences (31). Another prospective study of 10062 pregnant women reported that the risk of preterm birth did not increase among women who were positive for TPOAb and/or TgAb in early pregnancy, although premature rupture of membranes significantly increased (32). Importantly, thyroid autoantibodies are associated with the suboptimal development of offspring. One study evaluated the neurocognitive ability of 43 5.5-year-old children who were born at term. Children with TgAb-positive mothers had lower sensory performance and exercise scores, and those with TgAb-positive umbilical cord blood also had lower sensory performance scores (33). Although we do not know the pregnancy outcomes of women exposed to the COVID-19 outbreak who had isolated hypothyroxinemia or TgAb positivity in our study until writing this article, we will follow up on their pregnancy outcomes and the health status of their offspring.

The relationships between mood alteration and thyroid function were first recognized in a seminal review (34). Later, a meta-analysis of 20 studies found that individuals with anxiety were significantly more likely to have thyroid disease and that anxiety was inversely related to TSH levels (35). Several cross-sectional studies have shown that those with thyroid dysfunction have an increased risk of anxiety or depression (36–38). Therefore, previous studies have mostly focused on the relationship between depression or anxiety and TSH concentration, overt hyperthyroidism or overt hypothyroidism. The relationship between mental disorders and isolated hypothyroxinemia, FT3 or FT4 has not been explored. In this study, a higher concentration of FT3, a lower concentration of FT4, a higher risk of isolated hypothyroxinemia and a nonsignificantly higher concentration of TSH were found in pregnant women exposed to the COVID-19 outbreak. We speculate that this is because anxiety due to the COVID-19 outbreak amplifies the TSH enhancement of FT4 to FT3 conversion (39). This hypothesis requires further study.

In the current study, we found some interesting results in the sensitivity analysis. BMI was a determinant of thyroid function during pregnancy. Consistent with previous research (40, 41), we found that higher maternal BMI preconceptually was associated with higher FT3 concentrations, lower FT4 concentrations, and an increased risk for isolated hypothyroxinemia and TgAb positivity. The correlation between BMI and thyroid function has been reported previously and is hypothesized to be due to changes in energy balance caused by increased heat production in obese women (42). Why were the higher concentration of FT3, lower concentration of FT4 and higher risk of isolated hypothyroxinemia in women > age 40 exposed to COVID -19 outbreak not significantly different? First, the sample of pregnant women older than 40 years is relatively small. Furthermore, advanced maternal age was associated with lower FT3 (43). The enhancement of TSH on the conversion of T3 to T4 reaches its peak at the age of 30 to 40 years (44). Its enhancement begins to weaken after 40 (44). This can also explain why the risk of FT4 and isolated hypothyroxinemia is more significant in women aged 30 to 39 years in this study than in women < age 30. We also found that the higher maternal and paternal education level, the less significant the difference between the COVID-19 exposure and the nonexposure group, that is, the lower FT3 concentration, the higher FT4 concentration, and the lower risk of isolated hypothyroxinemia. A study that evaluated the depression and anxiety symptoms of pregnant women under COVID-19 may explain it. It showed that the higher the maternal education level, the less likely it is to report anxiety and depression because a higher education level often means higher income, more comprehensive knowledge of COVID-19, access to good medical resources and better prevention measures (21). Therefore, the thyroid function of pregnant women with higher education levels is less affected. The effect of paternal age on maternal FT3 and FT4 concentrations and the risk of isolated hypothyroxinemia is similar to that of maternal education level and age. This may be because people tend to choose those who are close to their age as their spouse. This can be proven in this study. The correlation coefficient between paternal age and maternal age was 0.72 (P<0.001). We also found that the main conclusions in TgAb-positive or TgAb-negative women are similar to the whole population. However, for TgAb-positive women, there was no significant difference in the increased risk of isolated hypothyroxinemia due to the COVID-19 outbreak. The small sample size of TgAb-positive women could partly explain this finding.

There are some limitations in our study. First, we did not demonstrate the mental health status of participants exposed to the COVID-19 outbreak. However, another study by our team, discussed above, evaluated the depression and anxiety symptoms of pregnant women in 25 hospitals in China before and at the time of the epidemic. It has been fully demonstrated that participants in this article are also more likely to experience symptoms of anxiety and depression under the pressure of the epidemic (21). Further, this study was completed within 2 months of the COVID-19 outbreak, and thus, only short-term thyroid responses were examined; long-term follow-up is required to examine subacute thyroid dysfunction, pregnancy outcomes, and postpregnancy thyroid function. Thyroid hormone changes were observed in pregnant women, and whether there is the same change in nonpregnant women and the general population is worthy of further research. In addition, although we have taken the confounding factors that affect thyroid hormone levels into account as comprehensively as possible, there are possibly some other unmeasured confounders causing bias in the propensity-score-matched cohort.

The current study provides evidence that exposure to extreme stress, such as the COVID-19 outbreak, can alter thyroid function in early pregnant women. When a pregnant woman is exposed to emergency or highly stressful events, the thyroid function of pregnant women deserves concern. However, whether this monitoring and treatment would lead to safe and effective results needs further research.

## Conclusion

This is the first study to examine alterations in thyroid functioning among pregnant women in their first trimester who are exposed to extreme stress, such as the COVID-19 outbreak. We found that exposure to the COVID-19 outbreak was independently associated with a higher FT3 concentration, a lower FT4 concentration, a higher risk of isolated hypothyroxinemia and a lower risk of TgAb positivity in pregnant women during the first trimester in Shanghai. Based on our study, we can infer that anxiety induced by COVID-19 during early pregnancy significantly influenced maternal thyroid function. Timely thyroid function tests and psychological intervention programs are recommended to be considered for pregnant women during emergencies or extremely stressful events and warrant additional research. Furthermore, the association between pregnancy outcomes and COVID-19 outbreaks requires further exploration.

## Data Availability Statement

The original contributions presented in the study are included in the article/**Supplementary Materials**, further inquiries can be directed to the corresponding authors.

## Ethics Statement

The studies involving human participants were reviewed and approved by The International Peace Maternity and Child Health Hospital institutional review board. The patients/participants provided their written informed consent to participate in this study.

## Author Contributions

All authors contributed to the study conception and design. Material preparation, data collection and analysis were performed by TL, CZ, HZ, YW, and LC. The first draft of manuscript was written by TL. Review and editing were performed by C-LD, HH, and YTW. All authors commented on previous versions of the manuscript. All authors contributed to the article and approved the submitted version.

## Funding

This research is supported by the National Natural Science Foundation of China (82001571), Program of Shanghai Academic Research Leader (20XD1424100), Outstanding Youth Medical Talents of Shanghai Rising Stars of Medical Talent Youth Development Program, Science and Technology Innovation Fund of Shanghai Jiao Tong University (YG2019GD04), Program of "Jiaotong University Star" Medical-Industry Interdisciplinary Research Fund (YG2020YQ29), COVID-19 Prevention and Control Project of the International Peace Maternity and Child Health Hospital (2020COVID1903) (2020COVID1904) and Clinical Research Project of Shanghai Municipal Commission of Health and Family planning (20184Y0349). Ethics approval was obtained by the institutional review board (No. GKLW2019-51).

## Conflict of Interest

The authors declare that the research was conducted in the absence of any commercial or financial relationships that could be construed as a potential conflict of interest.
