# Supplementary material for: Thyroid Hormone Changes in Early Pregnancy Along With the COVID-19 Pandemic
Source: Front Endocrinol (Lausanne). 2020 Dec 7;11:606723. doi: 10.3389/fendo.2020.606723 (PMC7750518; doi:10.3389/fendo.2020.606723)
Supplement: Supplementary file 1 [file DataSheet_1.docx]

| **Table S1 Factors associated with subclinical thyroid diseases in the Propensity-Score-Matched Cohort.** | | | | | | | | |
| --- | --- | --- | --- | --- | --- | --- | --- | --- |
| **Subclinical thyroid diseases** | **Maternal age distribution**  **OR (P value)** | **Maternal educational level**  **OR (P value)** | **Maternal BMI distribution**  **OR (P value)** | **Weeks gestational**  **OR (P value)** | **Gravida**  **OR (P value)** | **Paternal age distribution**  **OR (P value)** | **Paternal educational level**  **OR (P value)** | **Cohort group**  **OR(P value)** |
| Subclinical hyperthyroidism | 1.58(0.43) | 1.12(0.78) | 0.71(0.55) | 1.34(0.25) | 1.25(0.18) | 0.41(0.005) | 0.85(0.67) | 1.59(0.17) |
| Subclinical hypothyroidism | 1.01(0.97) | 0.97(0.92) | 0.83(0.44) | 0.80(0.32) | 0.83(0.25) | 1.44(0.26) | 1.00(0.99) | 0.58(0.07) |
| Overt hyperthyroidism | 0.65(0.65) | 0.26(0.15) | 1.33(0.65) | 2.38(0.19) | 1.69(0.11) | 1.14(0.89) | 2.24(0.35) | 0.50(0.38) |
| Isolated Hypothyroxinemia | 1.08(0.71) | 0.91(0.66) | 2.03(<0.001) | 1.17(0.27) | 1.14(0.14) | 0.96(0.86) | 0.90(0.60) | 1.77(0.004) |
| Thyroglobulin antibody positivity | 1.04(0.82) | 0.92(0.62) | 1.17(0.17) | 1.09(0.47) | 1.08(0.29) | 0.81(0.20) | 1.04(0.79) | 0.58(<0.001) |
| Elevated T3 | 0.65(0.63) | 0.84(0.85) | 2.77(0.03) | 1.96(0.25) | 1.33(0.39) | 0.92(0.92) | 0.71(0.68) | 0.69(0.22) |
| Low T3 | 0.92(0.75) | 1.33(0.25) | 0.73(0.10) | 0.70(0.04) | 1.26(0.04) | 1.03(0.90) | 1.71(0.03) | 0.40(0.11) |

Table shows the univariate analysis of the found factors associated with subclinical thyroid disease. Maternal BMI distribution is associated with isolated hypothyroxinemia and elevated T3. Weeks gestational, Gravida and paternal educational level are associated with Low T3. Paternal age distribution is associated with subclinical hyperthyroidism. Cohort group (Group 1 vs Group 2) is associated with isolated hypothyroxinemia and thyroglobulin antibody positivity.

| **Table S2 Subgroup analysis of FT3 concentration in the Propensity-Score-Matched Cohort.** | | | |
| --- | --- | --- | --- |
|  | **Group 2**  **Median (Q1-Q3)** | **Group 1**  **Median (Q1-Q3)** | **P value** |
| All women | 5.7(5.2-6.2) | 5.2(4.8-5.5) | <0.001 |
| Maternal age distribution | |  |  |
| 18-29 | 5.75(5.2--6.2) | 5.2(4.9-5.5) | <0.001 |
| 30-39 | 5.6(5.2--6.1) | 5.2(4.8-5.5) | 0.001 |
| >39 | 5.9(5.5--6.35) | 5.35(4.9-6.2) | 0.10 |
| Maternal Body mass index distribution |  |  |  |
| Low | 5.7(5.2-6.1) | 5(4.6-5.3) | <0.001 |
| Normal | 5.6(5.2-6.2) | 5.2(4.8-5.5) | <0.001 |
| Overweight | 6(5.5-6.5) | 5.2(4.85-5.6) | <0.001 |
| Obesity | 5.9(5.525-6.75) | 5.5(5.1-5.8) | 0.006 |
| Maternal educational level | |  |  |
| Low | 5.75(5.3-6.2) | 5.3(4.9-5.8) | 0.01 |
| Middle | 5.2(4.9-5.7) | 5.1(4.8-5.5) | <0.001 |
| High | 5.5(5.1-6.1) | 5.2(4.8-5.5) | <0.001 |
| Paternal age distribution | |  |  |
| 18-29 | 5.8(5.3-6.3) | 5.1(4.9-5.5) | <0.001 |
| 30-39 | 5.6(5.2-6.2) | 5.2(4.8-5.5) | <0.001 |
| >39 | 5.65(5.15-6.15) | 5.15(4.85-5.7) | 0.002 |
| Paternal educational level | |  |  |
| Low | 5.8(5.3-6.2) | 5.1(4.65-5.65) | <0.001 |
| Middle | 5.7(5.2-6.2) | 5.1(4.8-5.5) | <0.001 |
| High | 5.6(5.1-6.3) | 5.2(4.8-5.5) | <0.001 |

Table shows the FT3 concentrations in each subgroup of two groups which are expressed as median and quartile.

| **Table S3 Subgroup analysis of FT4 concentration in the Propensity-Score-Matched Cohort.** | | | |
| --- | --- | --- | --- |
|  | **Group 2**  **Median (Q1-Q3)** | **Group 1**  **Median (Q1-Q3)** | **P-value** |
| All women | 12.8(12.1-13.8) | 13.2(12.4-14) | <0.001 |
| Maternal age distribution |  |  |  |
| 18-29 | 13.1(12.3-14) | 13.3(12.6-14) | 0.008 |
| 30~39 | 12.8(12-13.7) | 13.1(12.3-13.9) | <0.001 |
| >39 | 12.2(11.8-13.25) | 12.6(11.6-13.6) | 0.95 |
| Maternal Body mass index distribution |  |  |  |
| Low | 13.1(12.4-14) | 13.4(12.6-14.25) | 0.32 |
| Normal | 12.9(12.2-13.8) | 13.2(12.5-14.05) | <0.001 |
| Overweight | 12.5(11.6-13.4) | 12.8(12-13.6) | 0.23 |
| Obesity | 11.8(11.5-12.8) | 12.8(11.9-13.3) | 0.03 |
| Maternal educational level |  |  |  |
| Low | 12.55(11.9-13.85) | 12.8(12.5-13.8) | 0.12 |
| Middle | 12.8(12.1-13.7) | 13.1(12.4-13.9) | <0.001 |
| High | 13(12.2-14) | 13.35(12.4-14.15) | 0.07 |
| Paternal age distribution |  |  |  |
| 18-29 | 12.9(12.2-13.95) | 13.4(12.7-14.3) | 0.03 |
| 30-39 | 12.9(12.1-13.8) | 13.2(12.4-13.9) | 0.002 |
| >39 | 12.35(11.9-13.45) | 12.9(12.2-13.6) | 0.07 |
| Paternal educational level |  |  |  |
| Low | 12.8(11.9-13.7) | 12.8(12.4-13.8) | 0.27 |
| Middle | 12.8(12.1-13.8) | 13.2(12.4-14) | <0.001 |
| High | 13(12.2-13.9) | 13.2(12.3-14) | 0.14 |

Table shows the FT4 concentrations in each subgroup of two groups which are expressed as median and quartile.

| **Table S4 Thyroid Hormone Concentrations of TgAb-negative women in the Propensity-Score-Matched Cohort.** | | | |
| --- | --- | --- | --- |
|  | Group 1 | Group 2 | P value |
| Thyrotropin, mIU/L(median,95% range) | 1.24(0.04-4.10) | 1.31(0.02-3.74) | 0.374 |
| Free thyroxine 3, pmol/L(median,95% range) | 5.2(4.2-6.6) | 5.7(4.3-7.4) | <0.001 |
| Free thyroxine 4, pmol/L(median,95% range) | 13.2(11.0-16.6) | 12.9(10.8-16.8) | <0.001 |
| Total thyroxine 3, nmol/L(median,95% range) | 2.0(1.4-2.8) | 2.0(1.5-2.7) | 0.543 |
| Total thyroxine 4, nmol/L(median,95% range) | 141.1(100.3-197.2) | 140.9(103.2-190.9) | 0.329 |

| **Table S5 Risk of (subclinical) thyroid diseases of TgAb-negative women in the Propensity-Score-Matched Cohort.** | | | | | |
| --- | --- | --- | --- | --- | --- |
| **(Subclinical) Thyroid diseases** | **Group 1 n(%)** | **Group 2 n(%)** | **Odds Ratio(95%)** | **Adjusted Odds Ratio(95% CI)** | **P value** |
| Subclinical hyperthyroidism | 9(1.5) | 20(3.1) | 2.08(0.94-4.60) | 2.08(0.94-4.60) | 0.07 |
| Subclinical hypothyroidism | 23(3.9) | 16(2.5) | 0.58(0.33-1.04) | 0.63(0.33-1.21) | 0.16 |
| Overt hyperthyroidism | 4(0.7) | 0(0) | 0 | 0 | 0.99 |
| Isolated Hypothyroxinemia | 38(6.5) | 71(11.1) | 1.81(1.20-2.73) | 1.77(1.16-2.69)a | 0.008 |
| Elevated T3 | 37(6.3) | 29(4.5) | 0.71(0.43-1.17) | 0.71(0.43-1.17)b | 0.18 |
| Low T3 | 5(0.8) | 1(0.2) | 0.18(0.02-1.67) | 0.17(0.02-1.44)a | 0.10 |

a: Maternal BMI is included as cofounding factor in logistic regression model; b: Maternal BMI, paternal education level, maternal age and age distribution are included as cofounding factor in logistic regression model

| **Table S6 Thyroid Hormone Concentrations of TgAb-positive women in the Propensity-Score-Matched Cohort.** | | | |
| --- | --- | --- | --- |
|  | **Group 1** | **Group 2** | **P value** |
| Thyrotropin, mIU/L(median,95% range) | 1.39(0.01-4.66) | 1.47(0.008-4.18) | 0.744 |
| Free thyroxine 3, pmol/L(median,95% range) | 5.0(4.1-6.2) | 5.6(4.1-9.5) | <0.001 |
| Free thyroxine 4, pmol/L(median,95% range) | 13.2(10.9-16.6) | 12.6(10.0-19.9) | 0.028 |
| Total thyroxine 3, nmol/L(median,95% range) | 2.0(1.4-2.8) | 2.0(1.4-3.0) | 0.733 |
| Total thyroxine 4, nmol/L(median,95% range) | 139.7(98.3-200.8) | 136.1(94.3-121.5) | 0.442 |

| **Table S7 Risk of (subclinical) thyroid diseases of TgAb-positive women in the Propensity-Score-Matched Cohort.** | | | | | |
| --- | --- | --- | --- | --- | --- |
| **(Subclinical) Thyroid diseases** | **Group 1 n(%)** | **Group 2 n(%)** | **Odds Ratio(95%)** | **Adjusted Odds Ratio(95% CI)** | **P value** |
| Subclinical hyperthyroidism | 5(3.6) | 2(2.3) | 0.63(0.12-3.30) | 0.63(0.12-3.30) | 0.58 |
| Subclinical hypothyroidism | 9(6.5) | 3(3.4) | 0.51(0.14-1.95) | 0.33(0.08-1.40)a | 0.13 |
| Overt hyperthyroidism | 0(0) | 2(2.3) |  |  | 0.99 |
| Isolated Hypothyroxinemia | 12(8.7) | 13(14.9) | 1.85(0.80-4.25) | 1.93(0.80-4.64)b | 0.14 |
| Elevated T3 | 13(9.4) | 6(6.9) | 0.71(0.26-1.95) | 0.71(0.26-1.95) | 0.51 |
| Low T3 | 0(0) | 1(1.1) |  |  | 0.99 |

a: Maternal age distribution and Gravida is included as cofounding factor in logistic regression model; b: Maternal BMI and BMI distribution, maternal age and weeks gestational are included as cofounding factor in logistic regression model


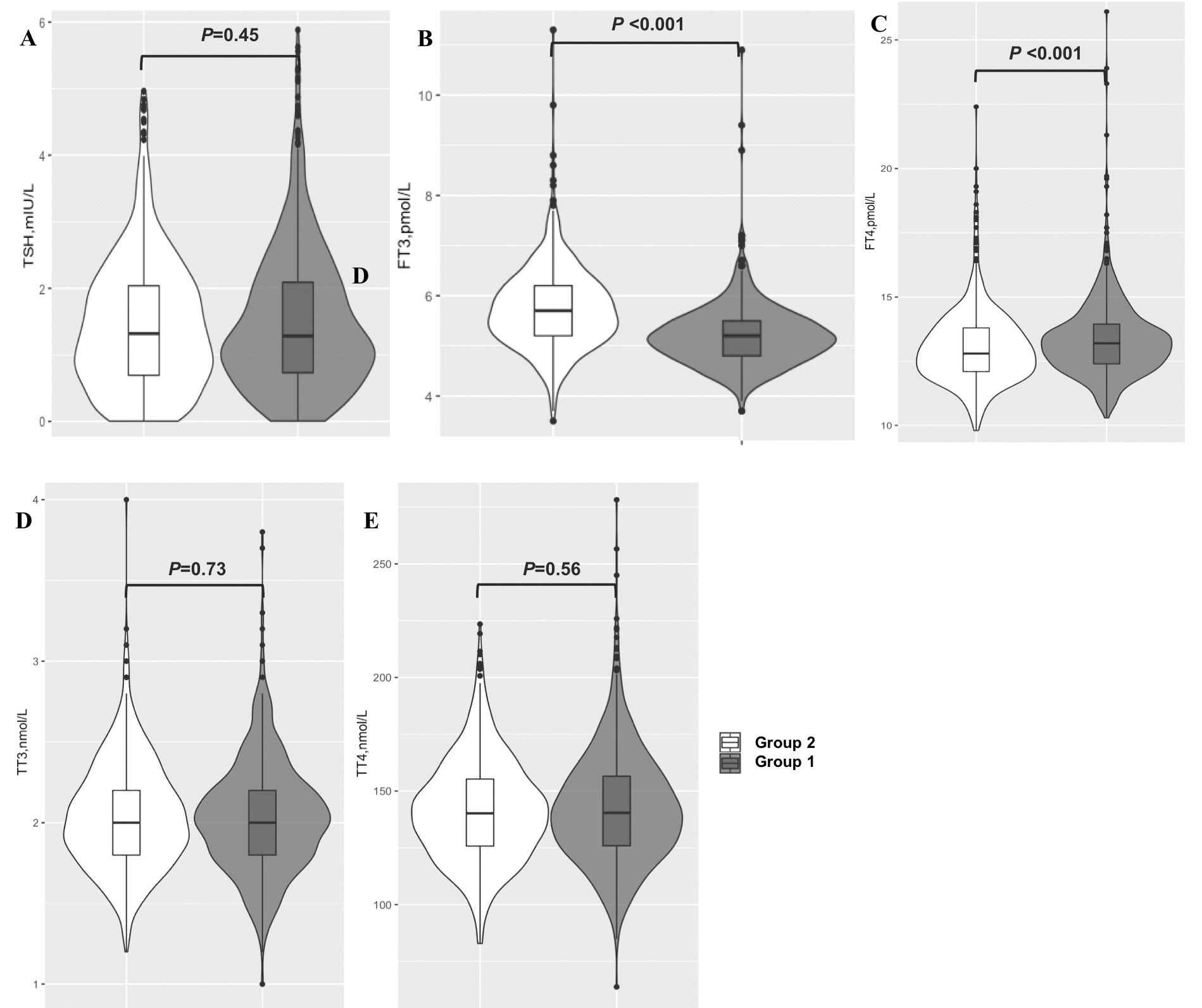


**Figure S1 Thyroid Hormone Concentrations in the Propensity-Score-Matched Cohort.** Figure shows COVID-19 outbreak was associated with significantly higher and lower FT4 concentration. The violin diagram is a combination of box plot and density plot. The thick black bar in the middle of the boxplot represents the median and quartile range, and the black line extending up and down represents the 95% confidence interval. The black dots on the black line indicate data values that exceed the 95% confidence interval. The outer contour indicates the distribution of the data. A. TSH concentration B. FT3 concentration C. FT4 concentration D. TT3 concentration E. TT4 concentration


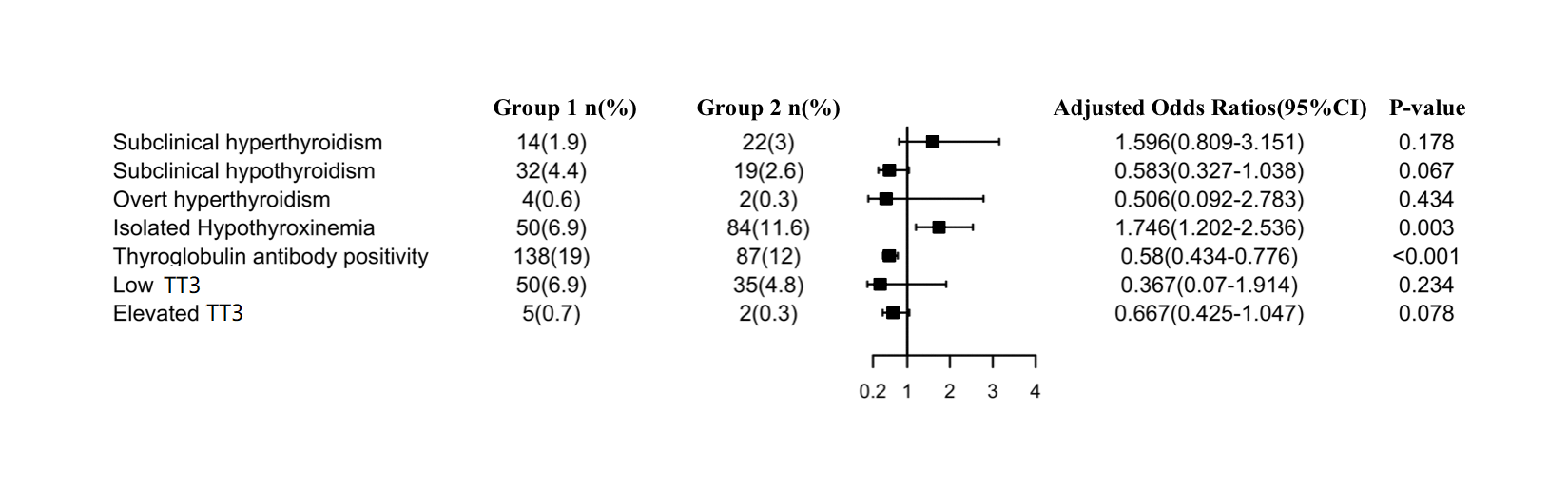


**Figure S2 Risk of (subclinical) thyroid disease in the Propensity-Score-Matched Cohort.** Figure shows the proportion of pregnant women with (subclinical) thyroid disease in Group 1 and Group 2 (expressed as the number of pregnant women with (subclinical) thyroid disease / the total number of women in certain group*100%). The forest chart in the fourth column describes the adjusted odds ratio in each subgroup. The diamond represents the overall adjustment odds ratio. The position of the small rectangle below indicates the point estimate of the adjustment odds ratio of each subgroup. The solid line range indicates the 95% confidence interval of the adjustment risk ratio of each subgroup. Adjusted odds ratio is calculated in the multivariate logistic regression model by including all confounding factors with a P value <0.1 in univariate analysis.
